# Lesion-symptom Mapping of Acceptability Judgments in Chronic Poststroke Aphasia Reveals the Neurobiological Underpinnings of Receptive Syntax

**DOI:** 10.1162/jocn_a_02134

**Published:** 2024-06-01

**Authors:** Danielle Fahey, Julius Fridriksson, Gregory Hickok, William Matchin

**Affiliations:** University of Montana; University of South Carolina; University of California, Irvine

## Abstract

Disagreements persist regarding the neural basis of syntactic processing, which has been linked both to inferior frontal and posterior temporal regions of the brain. One focal point of the debate concerns the role of inferior frontal areas in receptive syntactic ability, which is mostly assessed using sentence comprehension involving complex syntactic structures, a task that is potentially confounded with working memory. Syntactic acceptability judgments may provide a better measure of receptive syntax by reducing the need to use high working memory load and complex sentences and by enabling assessment of various types of syntactic violations. We therefore tested the perception of grammatical violations by people with poststroke aphasia (*n* = 25), along with matched controls (*n* = 16), using English sentences involving errors in word order, agreement, or subcategorization. Lesion data were also collected. Control participants performed near ceiling in accuracy with higher discriminability of agreement and subcategorization violations than word order; aphasia participants were less able to discriminate violations, but, on average, paralleled control participants discriminability of types of violations. Lesion-symptom mapping showed a correlation between discriminability and posterior temporal regions, but not inferior frontal regions. We argue that these results diverge from models holding that frontal areas are amodal core regions in syntactic structure building and favor models that posit a core hierarchical system in posterior temporal regions.

## INTRODUCTION

Syntax, or sentence structure, is a core feature of what fundamentally separates human language from other forms of communication. Accordingly, there has been great interest in and equally great debate about the neurological underpinnings of syntactic processing. Functional neuroimaging and intercranial studies have mostly converged on a broad frontal-temporal network of regions involved in various aspects of syntactic processing, including inferior frontal, inferior parietal, and anterior and posterior temporal lobe regions, although these studies have not yet been able to clearly isolate frontal from temporal contributions to sentence integration (for meta-analyses on fMRI studies, see Zaccarella, Meyer, Makuuchi, & Friederici, 2017; Rodd, Vitello, Woollams, & Adank, 2015; for intracranial studies, see Desbordes et al., 2023; Woolnough et al., 2023; Nelson et al., 2017). With respect to syntactic deficits in aphasia, there is also an impressive convergence. Damage or degeneration of inferior and middle frontal cortex has been consistently tied to expressive agrammatism, which is the reduction of syntactic complexity and omission of functional elements in speech production subsequent to stroke (i.e., expressive agrammatic aphasia) or resulting from neurodegeneration (i.e., the nonfluent/agrammatic variety of primary progressive aphasia; Matchin, Den Ouden et al., 2022; Matchin et al., 2020; Den Ouden et al., 2019; Sapolsky et al., 2010; Wilson et al., 2010; Goodglass, Christiansen, & Gallagher, 1993; Goodglass & Berko, 1960; Goodglass & Mayer, 1958). Most of the disagreement about the neurological bases of syntax stems from contradictory findings and claims in one major arena: the lesion basis of receptive syntactic deficits in aphasia.

The overwhelmingly dominant approach to assessing receptive syntactic deficits in aphasia is noncanonical sentence comprehension, established in a seminal study by Caramazza and Zurif (1976). Their logic was that participants with aphasia and a receptive syntactic deficit could nevertheless correctly match pictures to sentences with canonical or standard word order (e.g., “The lion that is scaring the baby is yellow,” p. 575) through a strategic heuristic of assuming that the first noun was also the agent of action. However, participants relying on this heuristic because of syntactic deficits would presumably be foiled by the presentation of sentences with noncanonical or nonstandard word order (e.g., “The lion that the baby is scaring is yellow”). Many studies have since used this approach to assess the extent to which people with aphasia (PWA) struggle with receptive syntax (Matchin, Basilakos et al., 2022; Matchin, Den Ouden et al., 2022; Kristinsson et al., 2019; Rogalsky et al., 2018; Pillay, Binder, Humphries, Gross, & Book, 2017; Mesulam, Thompson, Weintraub, & Rogalski, 2015; Thompson et al., 2013; Thothathiri, Kimberg, & Schwartz, 2012; Tyler et al., 2011; Wilson et al., 2010; Santi & Grodzinsky, 2007; Thompson & Shapiro, 2005; Grodzinsky, 2000; Grodzinsky & Finkel, 1998; Thompson, Lange, Schneider, & Shapiro, 1997). In contrast to the impressive convergence of results with respect to expressive agrammatism, there is much debate about which subtypes of patients will struggle with noncanonical sentence comprehension and the lesion correlates of these deficits. Receptive syntactic abilities have been associated with frontal areas (Mesulam et al., 2015; Thompson et al., 2013; Wilson, Galantucci, Tartaglia, & Gorno-Tempini, 2012; Wilson et al., 2010, 2011; Amici et al., 2007) or posterior temporal–parietal areas (Matchin et al., 2023; Matchin, Basilakos et al., 2022; Matchin, Den Ouden et al., 2022; Lukic et al., 2021; Matchin & Hickok, 2020; Den Ouden et al., 2019; Rogalsky et al., 2018). Still others have claimed that deficits result from a wide distribution of lesion patterns (Caplan et al., 2007; Dronkers, Wilkins, Van Valin, Redfern, & Jaeger, 2004; Dick et al., 2001; Caplan, Hildebrandt, & Makris, 1996).

There are at least two reasons for these discrepant reports. One is the fact that syntax is a multicomponent system. Different syntactic mechanisms are hypothesized to impair grammar in aphasia, which may thereby localize to different brain areas. Previous researchers have suggested that the paramount distinction with respect to the neurological organization of syntax is between what is often called syntactic movement, in which sentences are transformed by moving elements to other parts of the syntactic tree (Chomsky, 1980), and hierarchical phrase structure (Thompson et al., 2013; Friedmann, Reznick, Dolinski-Nuger, & Soboleva, 2010; Grodzinsky & Santi, 2008; Grodzinsky & Friederici, 2006; Thompson & Shapiro, 2005; Grodzinsky, 1986, 2000; Grodzinsky & Finkel, 1998; Gibson & Hickok, 1993). This hypothesis posits that receptive deficits in people with agrammatic Broca's aphasia and damage to Broca's area are thought to be restricted to movement, but that the basic hierarchical aspects of sentence structure are intact and processed in other areas. Much research has cast doubt on this particular hypothesis by demonstrating that people with agrammatism have difficulties with structures that seemingly do not require movement (Rogalsky et al., 2018; Rogalsky & Hickok, 2011; Wilson & Saygin, 2004; Hickok, 2000; Linebarger, Schwartz, & Saffran, 1983). In addition, the multicomponent nature of syntax should be stressed and explored more thoroughly, as it might explain some conflicting results in the literature. Second, the comprehension of complex, noncanonical sentences likely involves additional factors beyond syntactic processing, such as lexical access, semantic processing, and working memory. So, poor performance on tasks involving these sentences does not necessarily result from a syntactic deficit. For example, if phonological working memory deficits contribute to noncanonical sentence comprehension impairment, this may explain why inferior parietal and frontal areas have been implicated (Matchin & Hickok, 2020; Rogalsky & Hickok, 2011), whereas lexical and/or semantic deficits may explain why temporal lobe has been implicated.

An alternative approach to studying receptive syntactic ability is needed that can provide insights into this question, isolating potentially distinct domains of syntax. Syntactic acceptability judgment (SAJ) tasks, which ask participants to ascertain whether a sentence has appropriate syntax or not, target receptive syntactic knowledge more directly than complex noncanonical sentence comprehension tasks that have traditionally been used. Depending on the nature of the syntactic constructions used, SAJ tasks can also mitigate additional components recruited in processing complex noncanonical sentence comprehension, such as working memory confounds, as well as isolate different subdomains of syntactic processing.

In an influential early use of the SAJ paradigm, Linebarger and colleagues (1983) tested four people with Broca's aphasia who had been characterized as having agrammatic production and comprehension (i.e., worse performance on noncanonical relative to canonical structures). Participants were shown to have largely intact ability to perform fairly subtle SAJs across a wide range of sentence structures. In fact, a good deal of experimental work has shown that many people with agrammatic Broca's aphasia and deficits on noncanonical sentence comprehension mostly retain the ability to perform SAJs (Wulfeck, Bates, & Capasso, 1991; Wulfeck & Bates, 1991; Shankweiler, Crain, Gorrell, & Tuller, 1989; Linebarger et al., 1983). In contrast to the wealth of lesion-mapping research on receptive syntax using the noncanonical sentence comprehension paradigm, we are aware of only one study that has mapped the lesions related to deficits on SAJ. Wilson and Saygin (2004) performed lesion-symptom mapping (LSM) analyses on 16 people with chronic stroke-based aphasia. Participants completed an array of SAJs, involving fairly complex sentences with some quite subtle grammatical violations. Collapsing across sentence subtypes, results revealed that the posterior temporal lobe was most strongly implicated in overall SAJ deficits, regardless of patient subtype.

The present study used the SAJ paradigm to examine the ability of PWA to detect violations to three potentially distinct subdomains of syntax: word order, agreement (i.e., morphosyntactic inflections for syntactic features, such as plurality), and subcategorization (i.e., the specific preferences that words, chiefly verbs, have about the syntactic structures they appear in). The structures of interest are distinct from many prior studies in that we exclusively examined deviations in less complex syntax, with no syntactic movement or long-distance dependencies. We expected that participants would perform better at judging word order violations than the other conditions, in accordance with previous observations that word order violations are easier to detect than other types (Wulfeck & Bates, 1991; Wulfeck, 1988; Linebarger et al., 1983). We used *t* tests of *d*′ scores to determine whether groups differentially discriminated violations. We found that word order violations were easier to identify for both groups compared with other violation types and that control participants were significantly better at identifying violations than aphasia participants. With respect to the lesion correlates of syntactic violation discriminability, two dominant theories of syntax processing predict different lesion distributions associated with SAJ deficits. Models of syntactic processing in which the posterior temporal lobe plays a dominant role (Matchin & Hickok, 2020; Bornkessel-Schlesewsky & Schlesewsky, 2013) predict that poorer discriminability on each of the three subtypes of SAJ task to be primarily associated with damage to the posterior temporal lobe, whereas models of syntactic processing in which the inferior frontal lobe plays a dominant role (Mesulam et al., 2015; Wilson et al., 2010, 2012) predict damage associated with the inferior frontal lobe. Distributed models of syntactic processing (Caplan et al., 1996, 2007; Dronkers et al., 2004; Dick et al., 2001) predicted a wider distribution of lesions and would neither associate strongly with the posterior temporal lobe nor the inferior frontal lobe. We used voxel-based, LSM analyses on two primary ROIs, a frontal region comprising the left inferior frontal gyrus (IFG), pars triangularis and pars opercularis (respectively, IFGtri and IFGop), and a more posterior region that combined posterior superior and middle temporal gyri (pSTG/MTG). We also used whole-brain analyses to examine the lesion distribution associated with each violation. For all violation types, ROI analyses implicated only the posterior region, whereas whole-brain analyses implicated posterior portions of the temporal lobe, particularly the pSTG/MTG. Some portions of the IFG and insula were implicated in both agreement and subcategorization violations.

## METHODS

### Participants

Twenty-five right-handed PWA following left hemisphere stroke, at least 6 months poststroke, and 16 matched healthy controls participated in the study. PWA were recruited from speech-language-hearing clinics and aphasia support groups local to the researchers; control participants (controls) were recruited from patients' families and via snowball sampling. Exclusionary criteria (for both groups) consisted of hearing difficulties, dementia, head trauma, tumors, or multiple infarcts. Participants met via Zoom and were paid for their participation. Participants were fluent and dominant in English. PWA ranged in age from 44 to 80 years, with a mean of 62.4 years, and reported an average education of 15 years. These participants were diagnosed with left hemisphere vascular lesions at least 6 months before testing. PWA were classified through their Western Aphasia Battery–Revised Aphasia Quotient (WAB-R AQ) scores (Kertesz, 2006) as four patients were classified with anomia (*n* = 4), Broca's aphasia (*n* = 9), Wernicke's aphasia (*n* = 1), conduction aphasia (*n* = 3), global aphasia (*n* = 3), transcortical motor (*n* = 1), transcortical sensory/Broca's aphasia (*n* = 1), or not aphasic (*n* = 4).^1^ Control participants were matched on age and education. Control participants ranged in age from 56 to 76 years, with a mean of 63.7 years, and reported an average education of 17.5. The study was approved by the institutional review board at the University of South Carolina. Informed consent was obtained independently from all participants before their participation, with care taken to account for comprehension difficulties. Participants were consented with the observation of care partners, who were able to further communicate any questions by participants about task demands.

### Materials

Our study adapted the SAJ paradigm structure from Wulfeck and Bates (1991). Each participant was tested in two tasks, each with 64 sentences. Both tasks included three versions (Versions a, b, and c), which were randomly presented to participants to counterbalance sentence materials such that participants never saw both the grammatical and ungrammatical versions of the same sentence. Different versions included the same base sentences with alternating gender and number features in pronoun subjects (e.g., she/he/they) and alternating tense and aspect in verb constructions (e.g., is walking/was walking/have walked).^2^ Verb internal arguments were also counterbalanced for number. Only canonical, high-frequency verbs were selected. Half of the sentences in each task were licit in General American English,^3^ the other half ungrammatical. Ungrammatical sentences contained a single syntactic violation, manipulating either the verb phrase or its internal argument (i.e., either complement a prepositional phrase or noun phrase). In Task 1, half of the ungrammatical sentences contained a word order violation, and half contained an agreement violation. For each condition, there were 16 sentences with a violation, with 8 each on the verb and on the complement. In Task 2, the ungrammatical sentences contained a subcategorization violation of verbal complement type. Sentences in each condition were counterbalanced by whether complements were optional versus obligatory (e.g., “to agree with someone” vs. “to coach someone”). Selection of verbs and their complements followed normative data about subcategorization preferences (Connine, Ferreira, Jones, Clifton, & Frazier, 1984). Example sentences for each task and violation type are shown in Table 1A and B.

**Table 1.** Example Grammatical and Ungrammatical Sentences for Each of the Experimental Conditions

*(A) Sentence Conditions in Task 1*				
	*Word order*		*Agreement*	
	*Ungram*	*Gram*	*Ungram*	*Gram*
VP	They *talking were	They were talking	She *are baking	She is baking
	to some engineers	to some engineers	an apple pie	an apple pie

NP	He has listened	He has listened	They have baked	They have baked
	to *engineers some	to some engineers	*an apple pies	an apple pie

*(B) Sentence Conditions in Task 2*				
	*Optional*		*Obligatory*	
	*Ungram*	*Gram*	*Ungram*	*Gram*
VP	They *have hurrying to a meeting	They are hurrying to a meeting	They *have signaling to a boat	They have signaled to a boat

NP	He is answering *to a phone call	He is answering a phone call	She was chasing *in a cat	She was chasing a cat

(A) Task 1, which included the Word Order and Agreement conditions. (B) Task 2, which included the Subcategorization condition, divided into Optional and Obligatory subconditions. Gram = grammatical; Ungram = ungrammatical. The critical words signaling the grammatical violation are shown in **bold**. * Indicates an ungrammatical sentence.

Participants were presented recordings of the sentences that were created using Amazon Polly text-to-speech service (https://aws.amazon.com/polly/) with the “Joanna” voice in the Neural Text-to-Speech system. This produced automated speech that was determined to be indistinguishable from natural speech by several researchers, and without background noise or distortions. The output was an mp3 audio file at 24000 Hz with spoken sentences and associated metadata giving the word and sentence start times. The mp3 was converted to wav format using Audacity and cut using metadata with 500-msec lead and 800-msec lag time.

### Procedure

Participants were pseudorandomly assigned to begin with either Task 1 or 2, and to Versions a, b, or c; the same version was completed for each task. The experiment was presented using an in-house script developed with the Psychtoolbox extension (Kleiner et al., 2007; Brainard, 1997) in MATLAB (The MathWorks), which allowed randomization of items within task and version. Participants met study proctors via Zoom (https://zoom.us/) on a personal computer or tablet (but not cellular phone) with functioning microphones and cameras. Participants and proctors had microphones and cameras on during informed consent, but proctors muted cameras during task presentation. Instructions indicated that participants should focus on structural acceptability rather than prescriptive judgments. Each task began with eight practice sentences to familiarize participants with the procedure, as well as allow proctors to become familiar with participants' preferred response method. Practice items included grammatical and ungrammatical items; all but one ungrammatical practice item included multiple violations that would prevent aphasia participants being primed to detect violation types.^4^ Participants were allowed to hear each item one additional time upon request. Feedback was not provided except for during the practice session when proctors would indicate which response they were entering. Proctors shared computer audio to present items and manually recorded participants' responses as “grammatical” when participants believed that the sentence was well-formed, or “ungrammatical” when participants believed that sentences had a structural violation. Responses could be auditory or gestural (e.g., saying “good”/”bad” or displaying a thumbs up/thumbs down). Sessions were recorded, providing a back-up of manually entered results. Consistent feedback and instructions were provided to participants.

### Analyses

Analyses were performed on participants' *d*′ scores. *d*′ better reflects participants' sensitivity toward noticing violations, and less reflects participants' bias toward accepting responses (Haatveit et al., 2010). To calculate *d*′, we subtracted the *z*-transforms of false alarms (ungrammatical trials in which they said ungrammatical) from the *z*-transforms of hits (grammatical items trials in which they said grammatical; Macmillan & Creelman, 1991). To validate that aphasia participants performed significantly differently than control participants, *t* tests were conducted to identify differential discriminability by group to violations.

#### LSM

**Figure 1.**
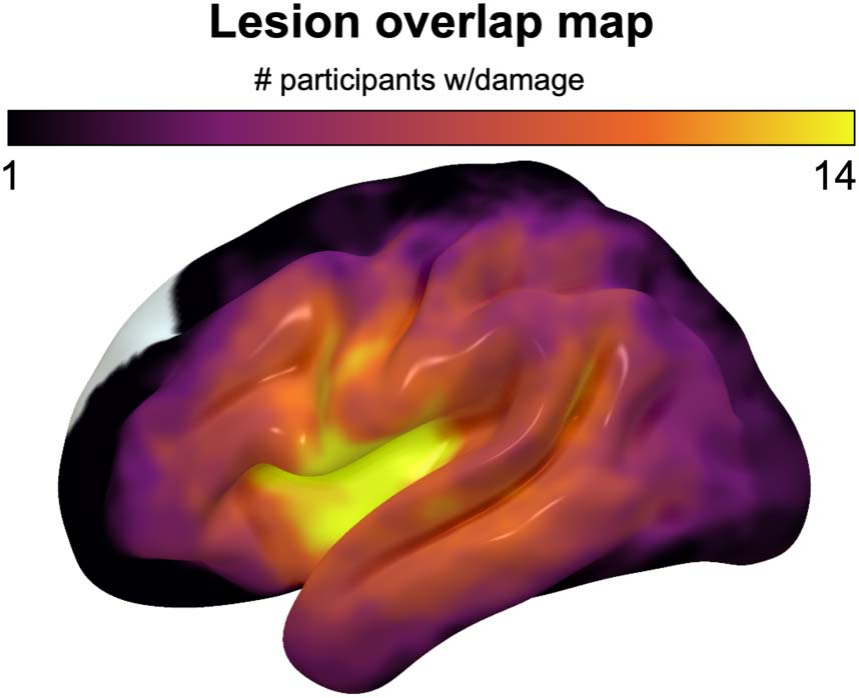
Lesion overlap map for participants with aphasia. Map represents all aphasia participants (*n* = 25), without one participant for whom MRI was contraindicated, with a maximum overlap of 14 individuals in a given region.

To gauge the relationship between our behavioral measures and damage to regions relevant to syntactic processing, we performed univariate ROI LSM analyses to identify the lesion distributions associated with each condition. LSM analyses were performed using *d*′ of participants' accuracy. LSM analyses utilized the *NiiStat* package in MATLAB (https://www.nitrc.org/projects/niistat/). Anatomical MRIs were collected for the participants at the McCausland Center for Brain Imaging. We mapped lesions using procedures and parameters from Matchin and colleagues (2020), demarcating lesions onto participants' T2 images by expert technicians or neurologists blind to the behavioral data. Participants' brains were aligned to standard space using SPM12. We used a binarized lesion mask for each participant and computed the number of voxels damaged in each region by dividing from the total number of voxels contained within that region. An overlap map of each participant's lesion^5^ included in final analyses are shown in Figure 1. To analyze the association between particular brain regions and grammatical comprehension, we created two ROIs by combining preexisting parcellations from the Johns Hopkins University atlas (Faria et al., 2012) as were used in Matchin and colleagues (2020). We utilized two regions, a frontal region labeled Broca's area that combined the left IFG, pars triangularis and pars opercularis (respectively, IFGtri and IFGop), and a temporal region that combined pSTG/MTG. We performed linear regression analyses in *NiiStat* (https://www.nitrc.org/projects/niistat/), calculating the percent damage to each ROI for each participant and relating these to each behavioral variable. We also performed whole-brain analyses using NiiStat, using an uncorrected individual voxel-wise threshold of *p* < .05. We corrected for multiple comparisons using permutation tests (10,000 permutations), and only voxels that were damaged in at least five participants (∼20% of sample) were included in whole-brain analyses. We supplemented the ROI analyses with exploratory uncorrelated, voxel-wise, whole-brain analyses to further inform the lesion distribution associated with each measure.

## RESULTS

### Behavioral Data

**Figure 2.**
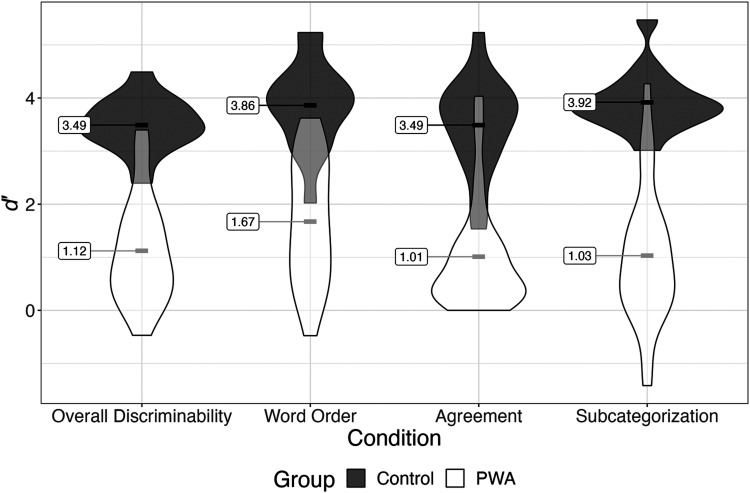
*d*′ scores of violations for PWA and controls. *d*′ scores reflect participants' sensitivity toward noticing violations. Higher scores reflect better discrimination of ungrammatical from grammatical items. Controls outperformed PWA across all conditions, but neither group performed fully at ceiling.

SAJ performance by group, task, and item type is summarized in Figure 2. Detailed demographic information and performance for aphasia participants are displayed below in Table 2. *t-*Test results revealed that control participants performed significantly better than aphasia participants for each measure examined. Between violation types, participants were better able to discriminate word order than agreement violations, and word order than subcategorization violations, with no significant difference between discrimination for agreement and subcategorization violations (a summary of comparisons is available in Table 3). As had been found in prior research (Wilson & Saygin, 2004; Wulfeck & Bates, 1991; Shankweiler et al., 1989; Linebarger et al., 1983), descriptive statistics showed that all participants performed more poorly on ungrammatical than grammatical items.

**Table 2.** Participants' Demographic Data, and Task and Violation Type Scores

*Participant*	*Demographics*				*Overall*			*Task 1 Scores*					*Task 2 Scores*		
	*Aphasia Type*	*WAB-R AQ*	*Current Age*	*Yrs. of Ed.*	*FA*	*H*	*d′*	*FA*	*Agreement Violations*		*Word Order Violations*		*FA*	*Subcategorization Violations*	
									*H*	*d′*	*H*	*d′*		*H*	*d′*
SAJ001	Anomic	84.2	75	16	0.02	0.73	2.78	0.00	0.44	2.58	0.81	3.62	0.03	0.84	2.87
SAJ002	Not aphasic	95.6	78	18	0.13	0.72	1.73	0.13	0.81	2.04	0.88	2.30	0.13	0.59	1.39
SAJ003	Wernicke's	67.8	59	16	0.48	0.75	0.71	0.50	0.56	0.16	0.94	1.53	0.47	0.75	0.75
SAJ004^‡^	Transcortical sensory	57.8	64	12	0.22	0.23	0.05	0.22	0.25	0.10	0.31	0.29	0.22	0.19	−0.11
SAJ005	Broca's	57.5	59	16	0.39	0.55	0.40	0.41	0.56	0.39	0.63	0.56	0.38	0.50	0.32
SAJ006	Broca's	77.4	64	16	0.23	0.78	1.50	0.25	0.56	0.83	0.99	3.17	0.22	0.78	1.55
SAJ007	Transcortical motor	78.2	61	12	0.14	0.42	0.88	0.19	0.38	0.57	0.75	1.56	0.09	0.28	0.74
SAJ008	Broca's	36.3	70	16	0.17	0.75	1.62	0.22	0.69	1.27	0.99	3.27	0.13	0.66	1.55
SAJ009	Not aphasic	99.2	63	16	0.03	0.91	3.18	0.03	0.94	3.40	0.81	2.75	0.03	0.94	3.40
SAJ010	Not aphasic	96.6	56	16	0.08	0.77	2.14	0.09	0.31	0.83	0.94	2.85	0.06	0.91	2.85
SAJ011	Anomic	90.1	66	16	0.14	0.44	0.92	0.22	0.38	0.46	0.63	1.10	0.06	0.38	1.22
SAJ012	Global	23.6	51	16	0.17	0.23	0.22	0.22	0.31	0.29	0.31	0.29	0.13	0.16	0.14
SAJ013	Broca's	64	66	18	0.19	0.56	1.04	0.19	0.56	1.04	0.88	2.04	0.19	0.41	0.65
SAJ014	Broca's	31.4	63	12	0.00	0.00	0.00	0.00	0.01	0.24	0.01	0.24	0.00	0.00	0.00
SAJ015	Global	31.4	52	16	0.22	0.34	0.37	0.22	0.38	0.46	0.50	0.78	0.22	0.25	0.10
SAJ016	Conduction	73.5	80	16	0.63	0.75	0.36	0.66	0.75	0.27	0.75	0.27	0.59	0.75	0.44
SAJ017	Broca's	35.5	74	13	0.36	0.20	−0.47	0.25	0.38	0.36	0.13	−0.48	0.47	0.16	−0.93
SAJ018	Global	5.6	59	16	0.08	0.11	0.19	0.06	0.06	0.00	0.38	1.22	0.09	0.00	−1.42
SAJ019	Not aphasic	96.8	44	18	0.03	0.94	3.40	0.06	0.99	4.03	0.88	2.68	0.00	0.94	4.27
SAJ020	Conduction	72.7	72	14	0.25	0.56	0.83	0.16	0.56	1.17	0.81	1.90	0.34	0.44	0.24
SAJ021	Anomic	85.3	51	16	0.09	0.58	1.52	0.16	0.56	1.17	0.81	1.90	0.03	0.47	1.78
SAJ023	Broca's	44.5	46	12	0.11	0.58	1.43	0.06	0.38	1.22	0.94	3.07	0.16	0.50	1.01
SAJ024*	Conduction	75.3	51	16	0.38	0.55	0.44	0.34	0.44	0.24	0.75	1.08	0.41	0.50	0.24
SAJ025	Broca's	71.7	70	14	0.11	0.75	1.90	0.09	0.63	1.64	0.94	2.85	0.13	0.72	1.73
SAJ026	Anomic	90.2	65	12	0.22	0.50	0.78	0.34	0.50	0.40	0.69	0.89	0.09	0.41	1.08
SAJ control001	–	–	76	14	0.02	0.92	3.57	0.03	0.94	3.40	0.99	4.36	0.00	0.88	3.88
SAJ control002	–	–	64	16	0.02	0.95	3.83	0.00	0.88	3.88	0.99	5.23	0.03	0.97	3.73
SAJ control003	–	–	66	15	0.02	0.94	3.69	0.03	0.99	4.36	0.99	4.36	0.00	0.88	3.88
SAJ control004	–	–	56	18	0.06	0.97	3.40	0.13	0.99	3.65	0.99	3.65	0.00	0.94	4.27
SAJ control005	–	–	68	20	0.02	0.83	3.10	0.03	0.81	2.75	0.88	3.01	0.00	0.81	3.62
SAJ control006	–	–	58	19	0.03	0.91	3.18	0.06	0.94	3.07	0.94	3.07	0.00	0.88	3.88
SAJ control007	–	–	61	21	0.02	0.97	4.02	0.00	0.99	5.23	0.99	5.23	0.03	0.94	3.40
SAJ control008	–	–	56	18	0.03	0.98	4.02	0.06	0.99	4.03	0.99	4.03	0.00	0.97	4.60
SAJ control009	–	–	68	19	0.06	1.00	4.49	0.13	0.99	3.65	0.99	3.65	0.00	1.00	5.47
SAJ control010	–	–	61	16	0.03	0.94	3.40	0.06	0.99	4.03	0.94	3.07	0.00	0.91	4.05
SAJ control011	–	–	57	18	0.05	0.97	3.54	0.06	0.99	4.03	0.99	4.03	0.03	0.94	3.40
SAJ control012	–	–	71	18	0.05	0.98	3.83	0.06	0.99	4.03	0.99	4.03	0.03	0.97	3.73
SAJ control015	–	–	63	16	0.05	0.95	3.35	0.09	0.94	2.85	0.99	3.82	0.00	0.94	4.27
SAJ control016	–	–	63	16	0.03	0.70	2.40	0.06	0.50	1.53	0.69	2.02	0.00	0.81	3.62
SAJ control017	–	–	68	18	0.05	0.88	2.83	0.09	0.75	1.99	0.99	3.82	0.00	0.88	3.88
SAJ control018	–	–	63	19	0.03	0.92	3.28	0.03	0.94	3.40	0.99	4.36	0.03	0.88	3.01

Aphasia type was determined from scores of Western Aphasia Battery; some participants' scores resulted in a diagnosis of “not aphasic.” (*) indicates that participant was contraindicated for MRI, so was excluded from LSM analyses. (‡) indicates that participant was unable to identify any grammatical violations in the experimental task; they were included in analyses nonetheless as performance on practice items indicated that they were able to understand the task. False alarms, hits, and *d*′ values are abbreviated FA, H, and *d*′, respectively.

**Table 3.** Comparisons of Group Level Accuracy in *t* Tests of *d*′ Scores between the Aphasia and Control Groups

		*df*	*t Statistic*	*p*
Overall Discriminability		38	−8.575	< .001
Task 1	Word order violations	38	−6.389	< .001
	Agreement violations	38	−7.656	< .001
Task 2	Subcategorization violations	38	−8.124	< .001

### ROI Analyses

**Figure 3.**
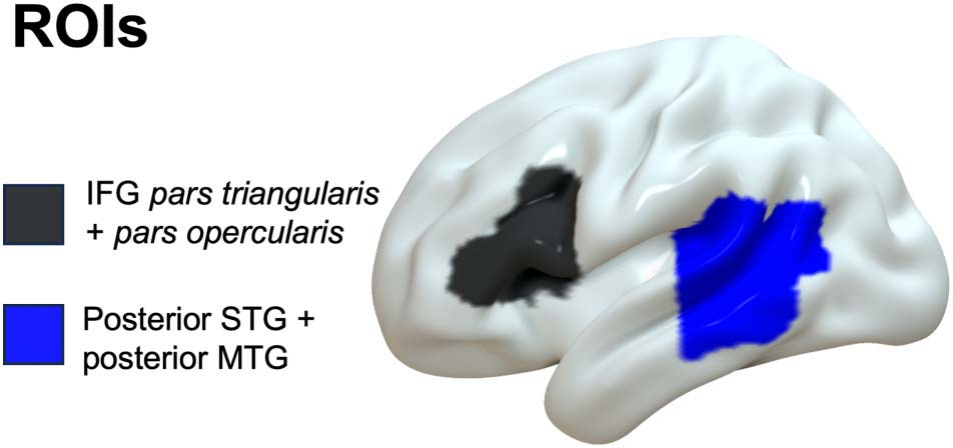
Reduced comprehension of grammatical violations for all three violation types (word order, agreement, and subcategorization) correlates to damage of the pSTG/MTG ROI, but none significantly correlates to the damage of Broca's area (the IFG). Our Broca's area ROI was defined as the posterior two-thirds of the IFG, including pars triangularis and pars opercularis and excluding pars orbitalis, following Tremblay and Dick (2016).

Figure 3 shows the ROIs we tested. We performed analyses both with and without a lesion volume covariate. Although lesion volume is an important factor in accurate lesion localization in whole-brain analyses (DeMarco & Turkeltaub, 2018), it also substantially reduces statistical sensitivity. Given that our ROI analyses are not testing precise localization but rather broad anatomical distinctions, we include both analyses here. We used a Bonferroni correction for multiple comparisons within each set of tests separately, using an adjusted alpha threshold of 0.05/3 = 0.0167 for each individual test. ROI analyses with lesion volume as a covariate showed some association with damage to the posterior temporal ROI (word order: *z* = −2.458, *p* = .014; agreement: *z* = −1.451, *p* = .147; subcategorization: *z* = −1.815, *p* = .070), although only the word order condition reached significance, but did not correlate with the inferior frontal ROI (word order: *z* = 1.033, *p* = .302; agreement: *z* = −.578, *p* = .563; subcategorization: *z* = .307, *p* = .759). ROI analyses without lesion volume covariate showed that grammatical violation comprehension was significantly associated with damage to the posterior temporal ROI for all three conditions (word order: *z* = −2.564, *p* = .010; agreement: *z* = −2.616, *p* = .009; subcategorization: *z* = −3.244, *p =*.001), but not to the inferior frontal ROI (word order: *z* = .307, *p* = .759; agreement: *z* = .307, *p* = .759; subcategorization: *z* = −1.793, *p* = .073).

### Whole-brain Analyses

**Figure 4.**
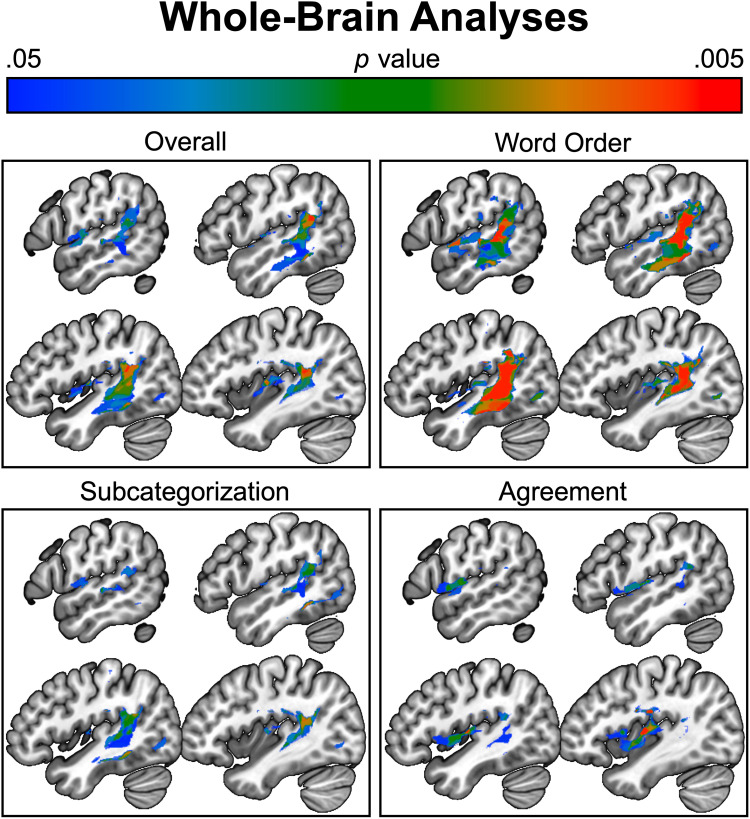
Whole-brain analyses (uncorrected) of three types of grammatical violation comprehension deficits.

Figure 4 shows results from the exploratory, uncorrected, whole-brain analyses (without lesion volume covariate). Permutation corrections for multiple comparisons revealed less than 10 voxels surviving overall ungrammatical or word order conditions, but 606 voxels in the agreement condition and 216 voxels in the subcategorization condition. Posterior portions of the temporal lobe, particularly the pSTG/MTG, were implicated in all three types of grammatical violation comprehension deficits, although this association was greatly reduced in corrected agreement violation deficits. Anterior portions of the parietal lobe were similarly, but weakly, associated to all three types of grammatical violation comprehension deficits as well. Portions of the IFG and insula were implicated in subcategorization and, more robustly, agreement violations, although were greatly reduced in corrected analyses of subcategorization violations. The wider semantic network, particularly the inferior temporal gyrus, posterior superior parietal lobe, and angular gyrus, was implicated in subcategorization and word order violation comprehension deficits.

## DISCUSSION

Our results showed that receptive syntactic ability, as assessed with SAJs, was at least mildly impaired in all participants with aphasia relative to age-matched controls. SAJ performance was also largely well above chance in the PWA group, consistent with previous research (Wilson & Saygin, 2004; Wulfeck & Bates, 1991; Shankweiler et al., 1989; Linebarger et al., 1983). LSM analyses found that overall deficits on the acceptability judgment task were associated with damage to the posterior temporal lobe ROI but not with damage to the Broca's area ROI, consistent with the LSM study on acceptability judgments reported by Wilson and Saygin (2004). This effect held for two of the three main structures examined separately (word order and agreement). Performance on the subcategorization task, although not significantly associated with damage to the Broca's area ROI, was trending toward significance, although this was only the case when lesion volume was not added as a covariate (see Matchin et al., 2023, for detailed discussion of the importance of lesion volume to the mislocalization of syntactic comprehension deficits in aphasia). These results generally converge with recent LSM studies of receptive syntactic ability using the more traditional noncanonical sentence comprehension measures, all of which have associated syntactic deficits primarily with damage to posterior temporal–parietal regions, but not frontal ones (Matchin, Basilakos et al., 2022; Matchin, Den Ouden et al., 2022; Kristinsson et al., 2019; Rogalsky et al., 2018; Thothathiri et al., 2012).

The whole-brain lesion analyses showed that performance across the three different syntactic domains largely converged on lesions to the posterior STS, specifically the posterior middle temporal gyrus including the ventral bank of the posterior STS. This region has been posited as critical to syntactic comprehension (Matchin & Hickok, 2020). However, there were suggestive differences in the lesion maps. The reduced threshold whole-brain LSM analysis of subcategorization showed a broader network than the other syntactic domains, including regions previously implicated in semantic processing, such as the angular gyrus and more anterior parts of the temporal lobe (Pylkkänen, 2019; Binder & Desai, 2011; Patterson, Nestor, & Rogers, 2007). One potential reason for this is that syntactic subcategorization is often co-mingled with semantic selection (Williams, 2015; Grimshaw, 1979, 1990). For example, a verb that selects a prepositional phrase argument (i.e., a syntactic preference) might also select a location argument (i.e., a semantic preference), making it difficult to disentangle these preferences. Agreement and subcategorization also implicated anterior insular cortex somewhat more than word order. Previous studies have shown that agreement processing in healthy participants recruits left inferior frontal lobe resources adjacent to anterior insula (Mancini, Quiñones, Molinaro, Hernandez-Cabrera, & Carreiras, 2017; Matchin, Sprouse, & Hickok, 2014; Moro et al., 2001) as has hierarchical processing (Bahlmann, Schubotz, & Friederici, 2008). The integration of the anterior insular cortex in the processing of syntactic comprehension might reflect broader integration of production-related regions in higher level syntactic processing (beyond simple phrase structure building), language-specific working memory, predictive mechanisms, or a combination of these (Matchin & Rogalsky, 2023; Matchin & Hickok, 2020; Matchin, 2018; Rogalsky et al., 2015; Fiebach, Schlesewsky, Von Cramon, & Friederici, 2004; Caplan & Waters, 1999). On the other hand, lesion studies often include insular damage which may be the result of its proximity to the left MCA (Hillis et al., 2004). Future studies examining the receptive ability of PWA to process different syntactic domains should account for these potential issues.

Another potential explanation for the dissociation of the three types of grammatical violation comprehension deficits is that they may correlate with aphasia type. To investigate this possibility, we examined whether the Broca's aphasia patients or the Wernicke's aphasia patient drove our results. First, we considered whether Broca's aphasia patients performed significantly differently from other patients using *t* tests. The results shown in Table 4 demonstrate that there were no significant behavioral differences. We did not perform similar tests with the Wernicke's aphasia patient, as there was only one in our cohort. Rather, we reran our ROI analyses without the Wernicke's aphasia patient. ROI analyses without the Wernicke's aphasia patient (without lesion volume as a covariate) showed stronger association with damage to the posterior temporal ROI than analyses that included them (word order: *z* = −2.606, *p* = .009; agreement: *z* = −2.422, *p* = .015; subcategorization: *z* = −3.282, *p* = .001), and similarly did not correlate with the inferior frontal ROI (word order: *z* = .238, *p* = .812; agreement: *z* = −1.589, *p* = .112; subcategorization: *z* = −1.391, *p* = .164). These results suggest that aphasia type is not a strong factor in our results. Although interest in effects from aphasia type on grammatical processing continue in the literature, recent reviews have suggested that aphasia type is not strongly related to lesion vasculature; therefore, it may not be a predictor of language deficits (Bunker & Hillis, 2022; Landrigan, Zhang, & Mirman, 2021). Additional research is needed to better gauge the relationship between aphasia type and lesion location or specific language deficits.

**Table 4.** Comparisons of Accuracy in *t* Tests of *d*′ Scores between the Broca's Aphasia Patients and All Other Aphasia Patients

		*df*	*t Statistic*	*p*
Overall Discriminability		23	0.640	.528
Task 1	Word order violations	23	−0.498	.623
	Agreement violations	23	0.432	.670
Task 2	Subcategorization violations	23	0.781	.443

Our results are also broadly consistent with functional neuroimaging studies of syntactic comprehension in healthy participants. Many functional neuroimaging studies have found structural effects in both posterior temporal cortex and inferior frontal cortex, among other regions (for meta-analyses, see Zaccarella et al., 2017; Fengler, Meyer, & Friederici, 2016; Rodd et al., 2015; Hagoort & Indefrey, 2014). However, some studies have only found structural effects in posterior temporal lobe and not frontal cortex (Matar, Dirani, Marantz, & Pylkkänen, 2021; Flick & Pylkkänen, 2020; Brennan, Stabler, Van Wagenen, Luh, & Hale, 2016; Bozic, Fonteneau, Su, & Marslen-Wilson, 2015; Tyler, Stamatakis, Post, Randall, & Marslen-Wilson, 2005; Tyler, Bright, Fletcher, & Stamatakis, 2004). Consequently, some researchers have suggested that the pIFG primarily supports working memory, prediction, and/or cognitive control in syntactic comprehension processing (Matchin & Rogalsky, 2023; Matchin, 2018; Matchin, Hammerly, & Lau, 2017; Rogalsky et al., 2015; Jakuszeit, Kotz, & Hastings, 2013; Wright, Stamatakis, & Tyler, 2012; Rogalsky & Hickok, 2011; January, Trueswell, & Thompson-Schill, 2009; Novick, Trueswell, & Thompson-Schill, 2005; Fiebach et al., 2004). Furthermore, this supporting role may be more fundamental to linearization primarily in syntactic *production* (Matchin & Rogalsky, 2023; Matchin & Hickok, 2020), which would account for the irregularity with which frontal regions are identified in recent LSM studies of receptive syntax in aphasia. Such a distinction of the roles of frontal and posterior temporal cortices has led to increasing support for a production-comprehension distinction in syntactic structure building (Arvidsson, Torubarova, Pereira, & Udden, 2023; Giglio, Ostarek, Weber, & Hagoort, 2022; Matchin & Wood, 2020). In this distinction, the frontal cortex primarily supports production whereas the posterior temporal cortex supports both comprehension and production (regarding a similar organization at the phonological level see also, Hickok, 2012; Hickok & Poeppel, 2007; Price et al., 1996; Wernicke, 1874). The issue of syntactic comprehension may hinge upon a delicate distinction between general working memory confounds versus language-specific processes that are difficult to disambiguate. Numerous suggestions have been proposed for the relationship between Broca's area to the psycholinguistic processes associated that decouple structure-building from structural memory and structural prediction, such as a morphosyntactic loop or language-specific working memory (Matchin & Hickok, 2020; Rogalsky, Matchin, & Hickok, 2008; Fiebach et al., 2004; Caplan & Waters, 1999). Although we attempted to reduce working memory confounds in the present study, it is impossible to completely eliminate these demands. To better distinguish syntax-specific resources like memory, future research would be benefited by adding a metric of working memory complexity or syntactic complexity.

Present whole-brain analyses differed from some studies of noncanonical sentence comprehension with respect to the specific location implicated within the posterior temporal lobe. Our overall SAJ deficits highlighted a somewhat more posterior locus than has been previously identified in some (Matchin et al., 2023; Maran, Numssen, Hartwigsen, & Zaccarella, 2022; Matchin, Basilakos et al., 2022; Matchin, Den Ouden et al., 2022; Murphy et al., 2022; Artoni et al., 2020) but not all (Lukic et al., 2021; Den Ouden et al., 2019; Thothathiri et al., 2012) studies. Specifically, our results implicate sulcal portions of the posterior middle temporal gyrus as well as more posterior regions than models of hierarchical lexical syntax (Matchin & Hickok, 2020). Prior studies of noncanonical sentence comprehension have used a broad variety of sentence subtypes and picture-matching paradigms, so the source of the variability is unclear. One possibility is that the posterior temporal complex implicated across syntactic comprehension research includes a number of loci for specific syntactic processes or tasks. Our results then may be understood as showing more syntax-sensitive loci than studies involving the processing of noncanonical or complex sentences. On the other hand, LSM studies are not as spatially precise as fMRI studies; thus, we hesitate to strongly interpret results beyond our ROIs. Future studies should more systematically investigate the contribution of task effects and sentence structure to these factors with respect to the precise localization of receptive syntactic deficits within the broader posterior temporal–parietal zone.

Finally, our study has a number of other limitations common to LSM studies. Age-related declines in syntactic abilities have been reported (Leckey & Federmeier, 2017; Tyler et al., 2010), potentially affecting the validity of our SAJ measures. However, our age-matched control participants, who were recruited to validate the experimental materials, performed strongly on the acceptability judgment measures. Our main goal was to identify the systematic lesion patterns it correlate with SAJ deficits, regardless if there is an additional overall effect of aging on performance. The improved rigor of this study concerns the measures and sample size for PWA, such that lesion mapping analyses would have improved validity and reliability. Future studies could recruit a greater number of participants from a broader age range to better account for effects of participant age and to more accurately localize lesion correlates (Lorca-Puls et al., 2018). For example, the addition of a lesion volume covariate to uncorrected whole-brain analyses with greater power may be relevant to the role of the insula, as damage to this region frequently covaries with damage to other areas. Relatedly, the improved power of additional participants may allow for interpretation of more precise ROIs, which is less viable without a lesion volume covariate. Furthermore, our participants were people with chronic stroke-based aphasia. Thus, it is possible that the brain networks uncovered in our LSM analyses do not reflect the same networks recruited in healthy participants because of reorganization and/or diaschisis. LSM studies with stroke patients particularly are limited by common lesion distributions affected by the vasculature. Nevertheless, we do note convergence of our results with other analyses of syntactic comprehension in acute stroke (Kristinsson et al., 2019) and in healthy populations more generally (see the Discussion section). It will be maximally informative to follow-up this study with a similar study on SAJs using TMS in healthy participants, acute aphasia, and other relevant populations.

In conclusion, our LSM study of SAJs in PWA converges with previous studies using noncanonical sentence comprehension as an indirect proxy for syntactic processing, providing support for the notion that the posterior temporal lobe (and not the inferior frontal lobe) is the most critical region supporting syntactic comprehension.

## Acknowledgments

We would like to thank the audiences of the Human Sentence Processing Conference in 2022, the Academy of Aphasia in 2021, the Society for the Neurobiology of Language in 20201 for their feedback on this work. We would also like to thank Jeremy Yeaton for their assistance with data analysis.

Corresponding author: William Matchin, Department of Communication Sciences and Disorders, University of South Carolina, 915 Greene St, Discovery 1, Room 202D, Columbia, South Carolina 29208, United States, or via e-mail: matchin@mailbox.sc.edu.

## Data Availability Statement

Data will be made available upon reasonable request by contacting the corresponding author.

## Author Contributions

Danielle Fahey: Conceptualization; Formal analysis; Investigation; Methodology; Project administration; Visualization; Writing—Original draft; Writing—Review & editing. Julius Fridriksson: Funding acquisition; Resources; Writing—Review & editing. Gregory Hickok: Conceptualization; Funding acquisition; Methodology; Writing—Review & editing. William Matchin: Conceptualization; Formal analysis; Funding acquisition; Investigation; Methodology; Project administration; Resources; Visualization; Writing—Original draft; Writing—Review & editing.

## Funding Information

National Institute on Deafness and Other Communication Disorders (https://dx.doi.org/10.13039/100000055), grant number: DC014664. Arnold School of Public Health, University of South Carolina (https://dx.doi.org/10.13039/100018427).

## Diversity in Citation Practices

Retrospective analysis of the citations in every article published in this journal from 2010 to 2021 reveals a persistent pattern of gender imbalance: Although the proportions of authorship teams (categorized by estimated gender identification of first author/last author) publishing in the *Journal of Cognitive Neuroscience* (*JoCN*) during this period were M(an)/M = .407, W(oman)/M = .32, M/W = .115, and W/W = .159, the comparable proportions for the articles that these authorship teams cited were M/M = .549, W/M = .257, M/W = .109, and W/W = .085 (Postle and Fulvio, *JoCN*, 34:1, pp. 1–3). Consequently, *JoCN* encourages all authors to consider gender balance explicitly when selecting which articles to cite and gives them the opportunity to report their article's gender citation balance.

## Notes

1. We note that these individuals presented with mild language deficits despite scoring outside of the aphasic range on our WAB-R assessment; hence, we still refer to them as PWA despite scoring as “not aphasic” at the time of testing. These individuals were recruited as participants because of being clinically diagnosed with aphasia in the acute stage.2. Past perfective aspect was not utilized since there is no subject agreement in English.3. Care was taken so that the constructions in the violation condition did not resemble potentially licit sentences in dialects other than General American English. All sentences in the violation condition containing a plural pronoun subject and auxiliary “is” or “was” were replaced with singular pronoun subjects and corresponding “are” or “were”.4. Practice items also served as an informal comprehension measure, as this ensured that participants understood the objective of the task.5. Without 1 participant, for whom MRI was contraindicated
